# Comparison between the protective effect of applying sodium fluoride varnish alone and applying CO2 laser with it in permanent teeth.  In Vitro study

**DOI:** 10.12688/f1000research.171971.2

**Published:** 2026-07-05

**Authors:** Sham Mohamad Farouq Alkhateeb, Chaza Nader Kouchaji, Abdulrhman Ahmad Alkhaled, Omar Hamadah

**Affiliations:** 1 Higher Institute of Laser Research and Applications, Damascus University, Damascus, Syria; 2 Department of Pediatric Dentistry, Damascus University Faculty of Dentistry, Damascus, Syria; 3 Oral Medicine Department, Damascus University Faculty of Dentistry, Damascus, Syria

**Keywords:** Laser; Gas; Dental Enamel; caries; Fluoride.

## Abstract

**Background:**

Fluoride administration plays a critical role in the management and prophylaxis of dental caries. Research indicates that laser irradiation can enhance the uptake of fluoride into dental enamel, an effect that is particularly pronounced when used as an adjunct to topical fluoride treatments. The present study aimed to evaluate the impact of CO₂ laser irradiation on fluoride uptake in the enamel of permanent teeth.

**Methods:**

Six human upper premolars, extracted for orthodontic reasons, were selected for this study. The roots were separated from the crowns, and each crown was then sectioned mesiodistally to create mesial and distal halves. These halves were randomly assigned to either an experimental or a control group (n = 6 per group). All specimens received an application of 5% sodium fluoride (NaF) varnish. Subsequently, the samples in the experimental group were subjected to irradiation with a 1 W CO _2_ laser for a duration of 15 seconds. To assess the effects of the treatments, fluoride uptake was measured and the topographic characteristics of the enamel surface were examined using scanning electron microscopy (SEM) and energy-dispersive X-ray spectroscopy (EDX). Statistical analysis of the data was performed using SPSS software version 26 (IBM, USA).

**Results:**

Scanning electron microscopy (SEM) structural analysis revealed that the enamel surfaces in the fluoride alone group maintained a typical etched pattern with visible micropores. In contrast, the experimental group subjected to both sodium fluoride varnish and CO _2_ laser irradiation demonstrated distinct surface melting, fusing of enamel crystals, and a smoother recrystallized morphology. Energy-dispersive X-ray spectrometry analysis showed a significant increase in the percentage of fluoride with the application of the laser, The results of the mean ranks showed that the fluoride and CO₂ laser group recorded significantly higher ranks in the fluoride element (8.75 vs. 4.25), reflecting a higher concentration and confirmed statistical significance. Statistically, in the Mann-Whitney U test, a significant statistical difference in fluoride ratios appeared between the two groups (P value = 0.026) smaller than (0.05), which indicates the effectiveness of using CO₂ lasers in enhancing fluoride absorption within the tooth structure.

**Conclusion:**

The results showed CO₂ laser irradiation of dental enamel resulted in increased fluoride uptake.

## Introduction

Dental caries deserves attention because it has become a chronic disease affecting children worldwide. ^1^^,^^2^ Dental caries is a multifactorial disease caused by the simultaneous interaction of various factors— sugar and dental biofilms —in the oral cavity. The introduction of fluoride into dental practice over seventy years ago is widely regarded as the principal reason for the global decline in dental caries. Despite this public health success, the sole confirmed adverse effect of fluoride use in dentistry is dental fluorosis, a condition resulting from excessive fluoride ingestion during tooth development. Recent epidemiological trends indicate a concurrent rise in the prevalence of dental fluorosis and a decline in caries rates. ^3^

The most common varnish used in dentistry to prevent tooth caries is sodium fluoride (NaF). ^4^ Topical application of NaF on teeth mitigates enamel demineralization while enhancing the remineralization process. The primary antimicrobial mechanism of fluoride is not direct bacterial destruction, but rather an indirect action through the inhibition of bacterial acid production, which results in a higher pH at the tooth surface. ^5^^,^^6^

The combined use of laser irradiation and topical fluoride represents a novel approach to caries prevention by increasing the enamel's affinity for and incorporation of fluoride. Zancope et al. reported that CO _2_ lasers cause morphological changes on the surface of tooth enamel, such as fusion and dissolution. ^7^ Studies evaluating the effect of carbon dioxide laser on the structure of tooth enamel and dentin have shown high absorption by dental tissue. ^8^ Specifically, the 10,600 nm CO _2_ laser features an absorption coefficient in hydroxyapatite that allows specific depth of penetration and flow to produce the desired thermal effects on caries inhibition. ^9^

Although topical fluoride applications, such as varnishes, remain the gold standard in caries prevention, their clinical efficacy is often limited by their susceptibility to rapid dissolution in oral fluids, necessitating frequent reapplications. However, repeated high-dose applications raise concerns regarding potential systemic toxicity and fluorosis. ^3^ To overcome these limitations and enhance the longevity of fluoride retention, modern preventive dentistry has focused on synergistic technologies. The CO _2_ laser (10,600 nm) has emerged as a promising modality; when irradiated at specific sub-ablative parameters, it induces microstructural and thermal alterations in the enamel surface. This photothermal effect temporarily increases enamel permeability and promotes the stable conversion of hydroxyapatite into a highly acid-resistant fluorapatite phase. ^10^ Therefore, the integration of laser therapy is proposed not to replace fluoride, but to optimize its chemical binding and depth of penetration, thereby potentially offering prolonged cariostatic protection with lower effective fluoride doses.

This study aimed to evaluate and compare the efficacy of CO _2_ laser irradiation versus a CO _2_ application in enhancing fluoride uptake by the surfaces of permanent teeth following treatment with NaF varnish.

## Materials and methods

### Source of teeth specimens

The six human upper premolars were obtained from the Department of Oral and Maxillofacial Surgery in College of Dentistry, University of Damascus after routine extractions for orthodontic reasons and not specifically for this study. Teeth were stored in distilled water at 4 °C until use.

**Figure 1.**
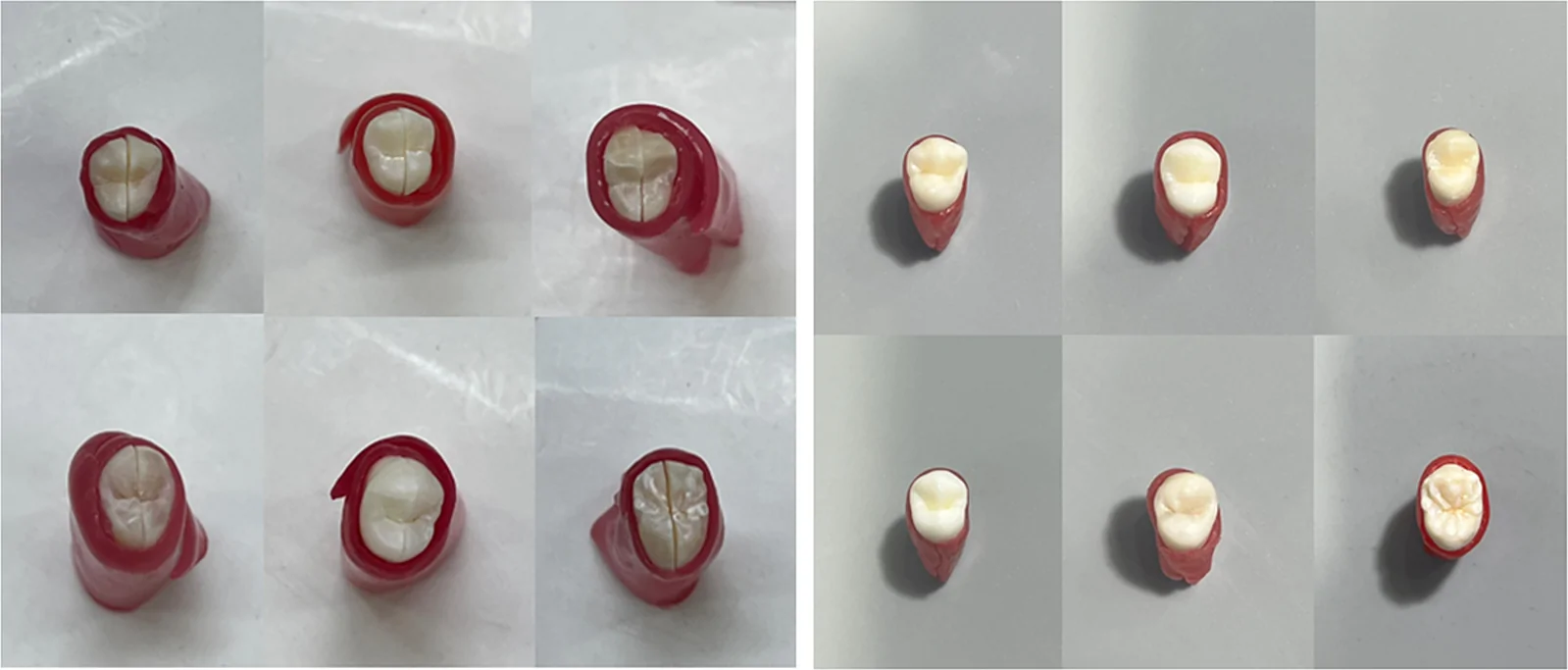
A picture of the teeth used in the research before and after the section.

Teeth were examined to ensure they were free of any caries, abrasions or fractures. They were divided into two sections, a mesial section and a distal section, so that each treatment was applied to a section of the tooth. The root was cut from the Cementoenamel junction and the crown were stored in distilled water to prevent dehydration ( Figure 1).

Mesial section was used as the experimental specimen and the distal as the control.

**Figure 2.**
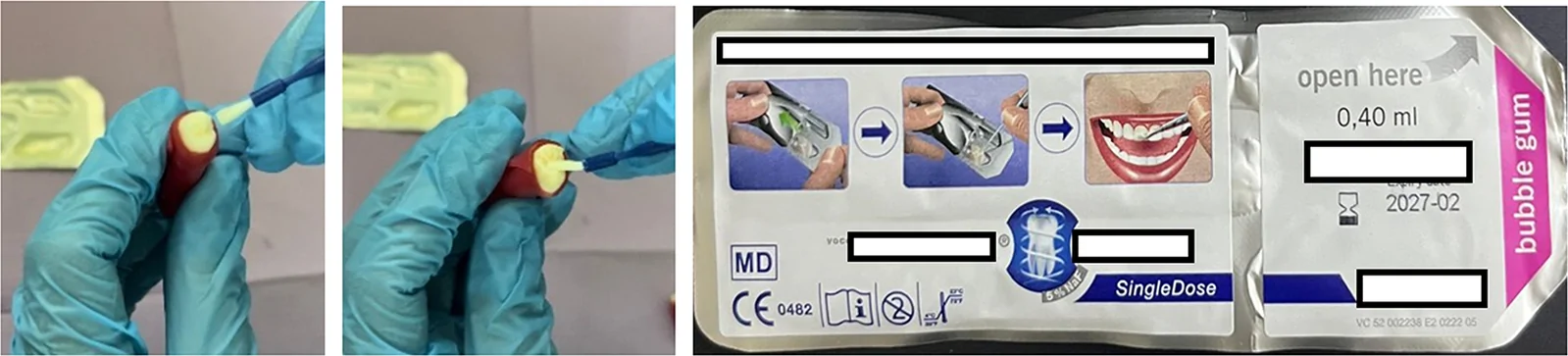
A picture of the sodium fluoride varnish used by VOCO and how it is applied.

All specimens were first dried and treated with 5% NaF varnish (VOCO®, Germany). A distinction was then made between the groups: the experimental specimens were laser irradiated, while the control specimens were not. Sodium fluoride varnish (5% NaF varnish (VOCO, Germany, Product Code: [190/10]) was applied to the mesial section of the tooth with a carbon dioxide laser with a wavelength of 10600 nm (power 1 W – pulse duration 15 ms) while the control sample was in the distal section and sodium fluoride varnish alone was applied ( Figure 2).

The varnish was applied using a small brush according to the manufacturer’s instructions by applying a thin layer over the entire occlusal surface of the tooth.

**Figure 3.**
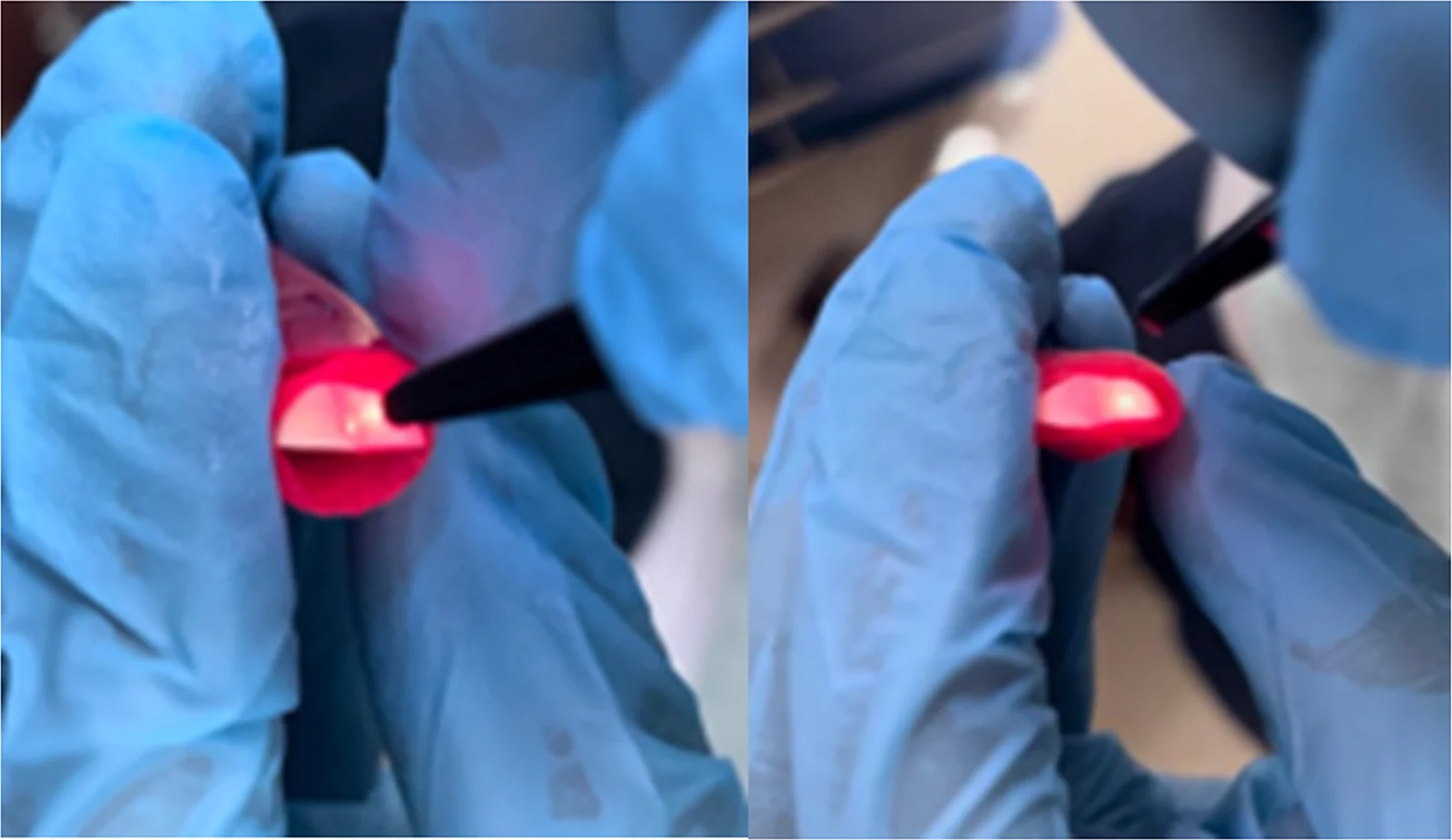
Image of the parameters used.

The sample in the mesial section was exposed to CO _2_ laser radiation (10600 nm wavelength, 1 W power, pulsed mode, 15 ms pulse duration) with a wavelength of 10600 nm and a power of 1 W for 15 seconds with a pulsed mode and a pulse duration of 15 ms ^11^ at a distance of 1 cm. Irradiation was performed in a continuous scanning motion that allowed the entire surface to be irradiated and we re-preserved it with distilled water ( Figure 3).

**Figure 4.**
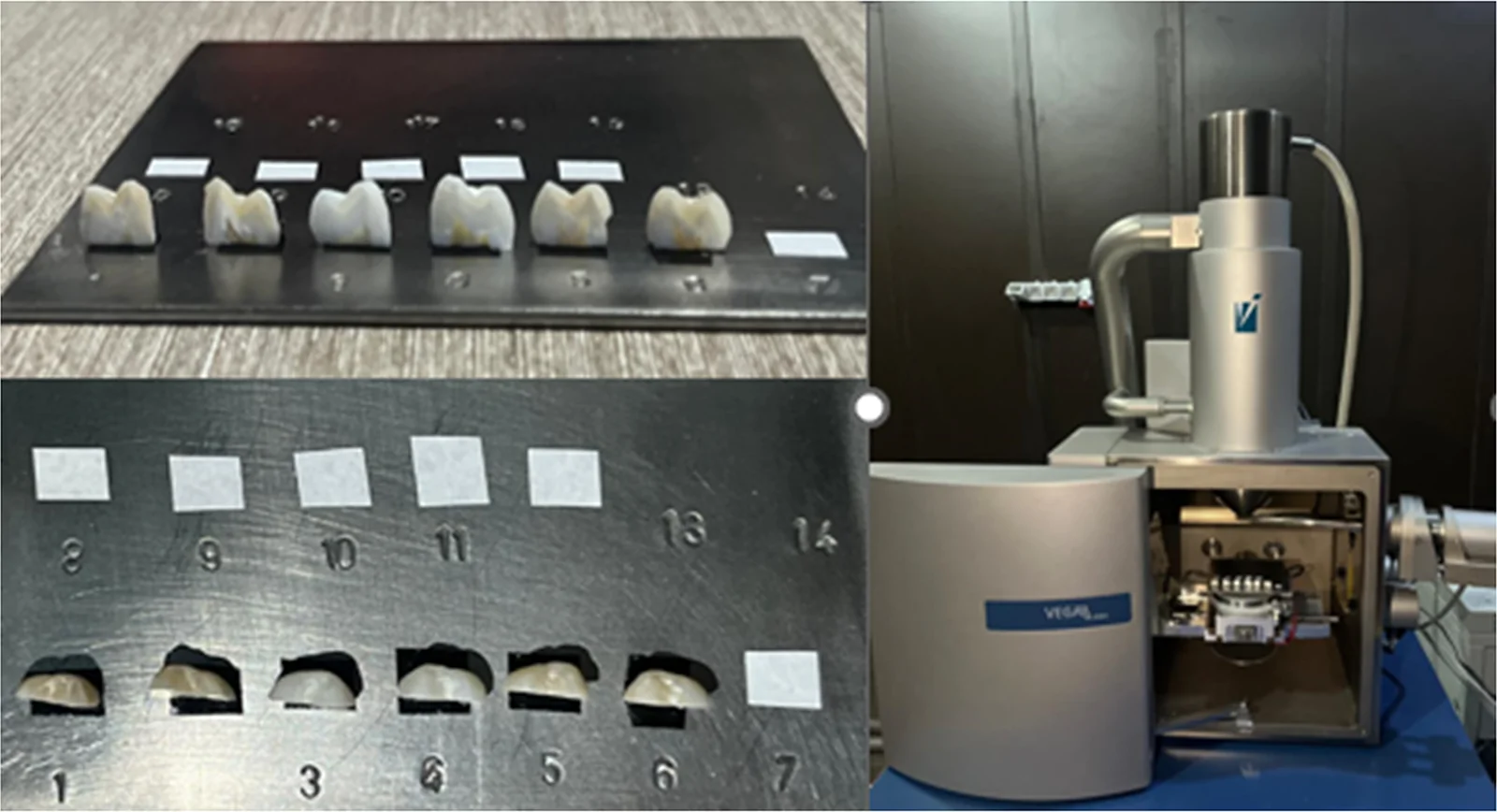
Image of the sample after preparation for entry into the scanning electron microscope.

The test of the effect of the laser on the varnish was evaluated using scanning electron microscopy (SEM) (T-Scan company VEGA II XMU, Czech Republic). The amount of fluoride, calcium, phosphorus, and carbon remaining on the specimens was tested using energy-dispersive X-ray spectroscopy (EDX) (Oxford Instruments, UK) attached to the microscope located in the Atomic Energy Commission ( Figure 4). The samples were analyzed by SEM to observe the topographic properties of the enamel surface after different treatments, and images of the central region were taken using two different detectors: Secondary Electrons (SE) and Backscattered Electrons (BSE). EDX analysis provided non-destructive quantitative data on the chemical composition and elemental distribution over the surface area. ^12^ The response variables evaluated were changes in the concentrations of fluoride, carbonate, calcium, and phosphorus that constitute the hydroxyapatite structure.

### Ethical approval and informed consent

This *in vitro* study utilized extracted human teeth collected from patients who underwent orthodontic treatment. This study was from 24/8/2025 to 27/9/2025 The study protocol was reviewed and approved by the Institutional Review Board (IRB) of College of Dentistry, University of Damascus On August 21, 2025 under reference number (DN-210-125-3-9-1/21/8/2025). All participants (or their guardians) provided written informed consent for the use of their extracted teeth for research purposes, in accordance with the principles of the Declaration of Helsinki. Patient identifiers were removed to ensure confidentiality.

## Results

The results were evaluated according to the surface shape in electron microscope images and according to the concentration of fluoride, calcium, phosphorus and carbon.

**Figure 5.**
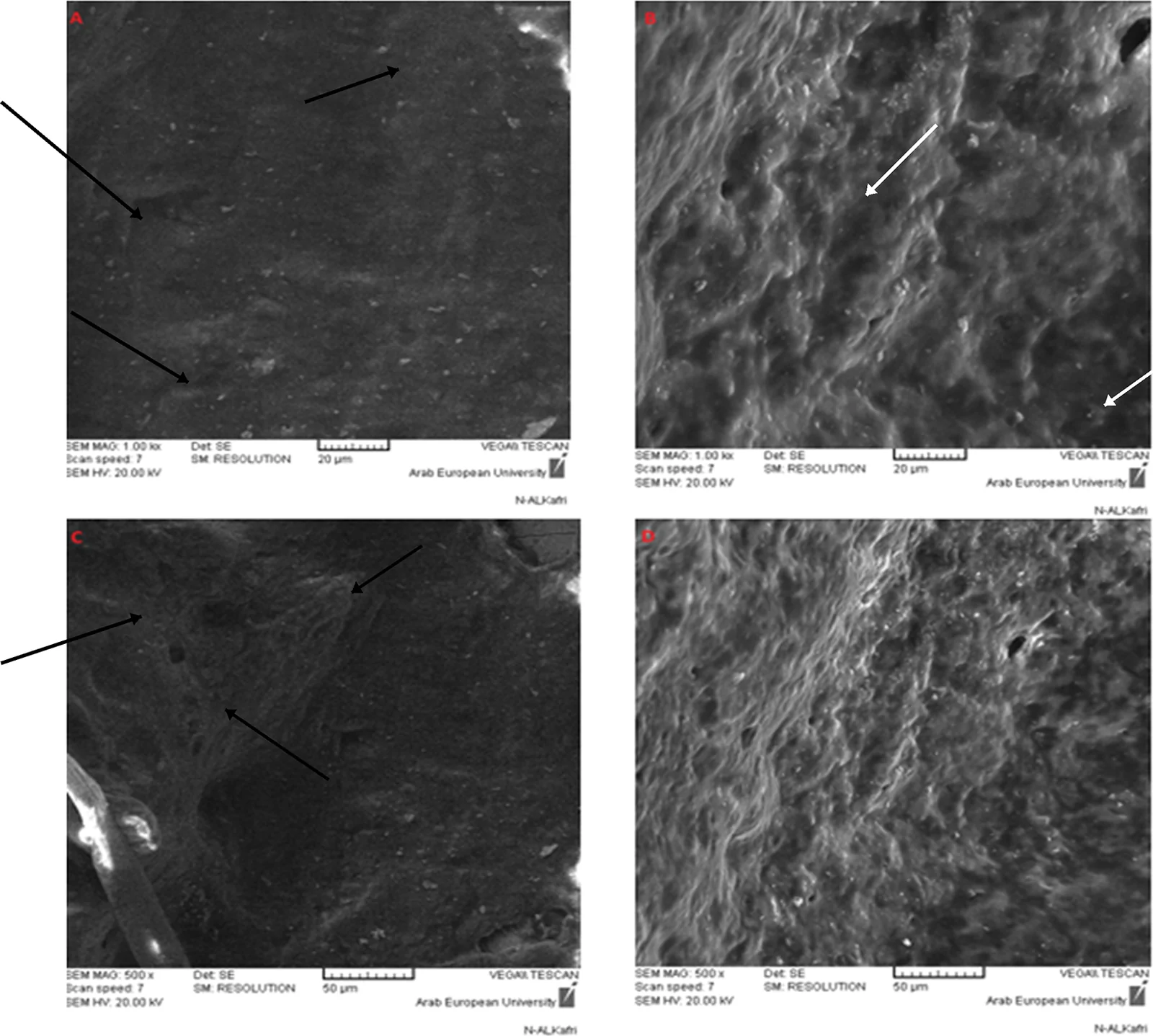
SEM micrographs of enamel surfaces. (A) Mesial section (fluoride + laser) using SE detector (×1000) : Black arrows indicate zones of superficial enamel melting and crystal fusion. (B) Distal section (fluoride only) using SE detector (×1000) : White arrows point to typical patent micropores. (C) Mesial section (fluoride + laser) at ×500 magnification: Black arrows highlight the smooth, recrystallized surface topography. (D) Distal section (fluoride only) at ×500 magnification showing unaltered surface morphology.

**Figure 6.**
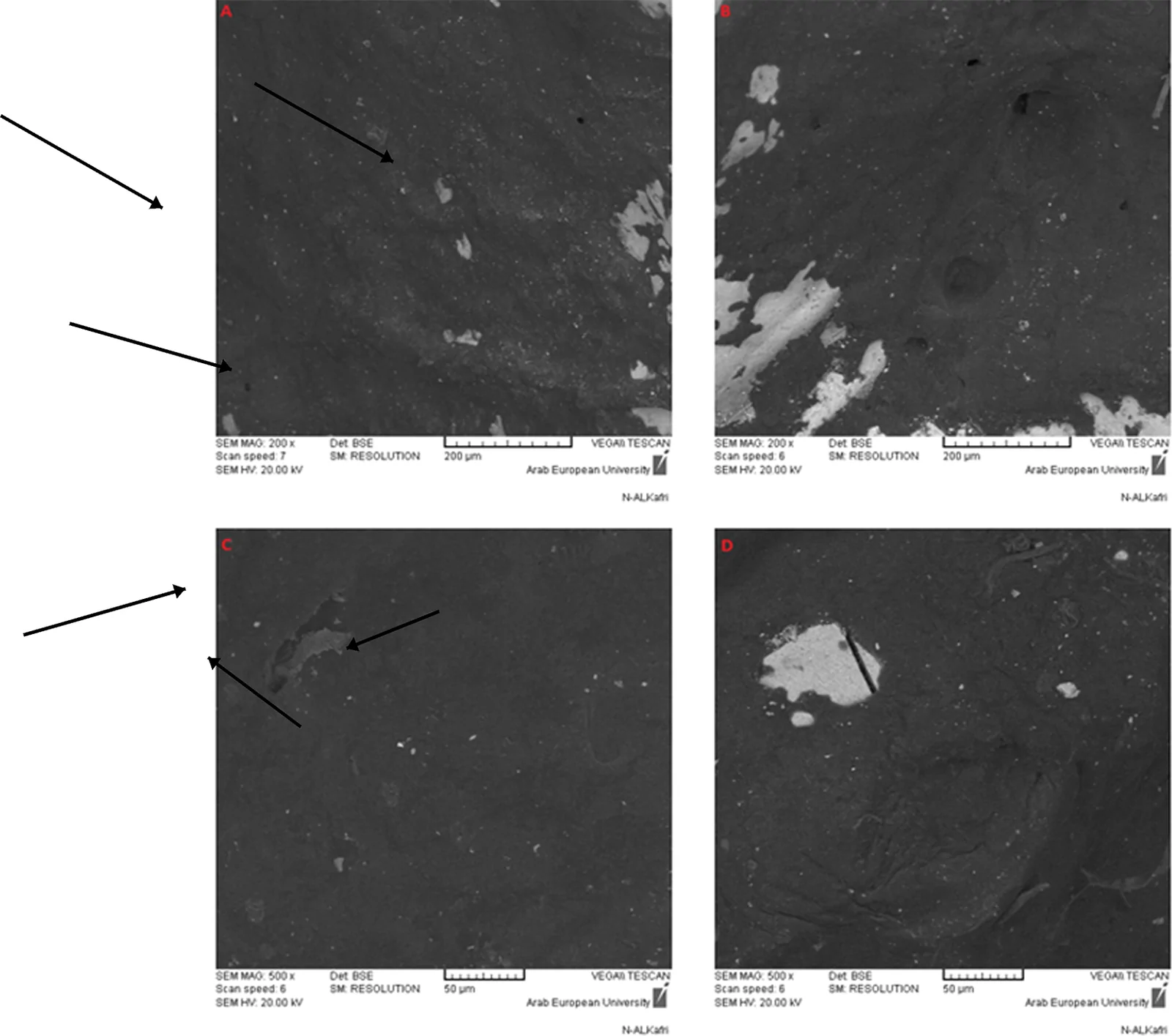
SEM micrographs of enamel surfaces using BSE detector. (A) Mesial section (fluoride + laser) at ×200 : Black arrows indicate the boundary of thermal modification and fused mineral deposits. (B) Lateral section (fluoride only) at ×200 showing baseline surface characteristics. (C) Medial section (fluoride + laser) at ×500 : Black arrows mark the homogeneous recrystallized hydroxyapatite lattice. (D) Lateral section (fluoride only) at ×500 displaying micro-roughness without melting features.

In this study, SEM images showed that CO _2_ laser irradiation fuses the surface, creating a smooth, recrystallized side. Fusion between enamel surface crystals was also observed after carbon dioxide laser treatment ( Figures 5 and 6).

Comparing the apparent differences in the mean of the measured items between the two groups ( Table 1) and the descriptive statistics for fluoride, calcium, phosphorus, and carbon ratios in both study groups ( Tables 2– 5).

**Table 1.** The comparison between elements using the mean.

Element	Fluoride group	Fluoride + CO _2_ laser	The difference between averages	Comments
Fluoride	0.35	1.73	+1.38	Obvious apparent increase in fluoride with laser application
Calcium	1.62	3.72	+2.10	Significant apparent increase in calcium
Phosphorus	0.99	0.65	−0.34	Slight decrease in phosphorus
Carbon	72.89	67.30	−5.59	Slight carbon reduction

**Table 2.** The descriptive statistics of fluoride ratios.

Fluoride	Fluoride group	Fluoride + CO _2_ laser group
**Mean**	0.4	1.7
**Std. Deviation**	0.3	1.4
**Median**	0.2	1.4
**Minimum**	0.1	0.2
**Maximum**	1.0	4.3

**Table 3.** The descriptive statistics of calcium ratios.

Calcium	Fluoride group	Fluoride + CO _2_ laser group
Mean	1.62	3.72
Std. Deviation	1.56	6.28
Median	1.10	1.28
Minimum	0.50	0.60
Maximum	4.65	16.50

**Table 4.** The descriptive statistics of phosphorus ratios.

Phosphorus	Fluoride group	Fluoride + CO _2_ laser group
Mean	0.99	0.65
Std. Deviation	1.13	0.92
Median	0.39	0.35
Minimum	0.11	0.00
Maximum	2.44	2.50

**Table 5.** The descriptive statistics of carbon ratios.

Carbon	Fluoride group	Fluoride + CO _2_ laser group
Mean	72.89	67.29
Std. Deviation	8.97	7.42
Median	74.24	68.75
Minimum	57.13	57.80
Maximum	84.12	77.56

**Table 6.** Mann-Whitney U test results.

	Fluoride	Calcium	phosphorus	Carbon
Test value	4.5	15	17	11
P value	0.026	0.699	0.937	0.31
Comparison with significance level	Less than 0.05	Greater than 0.05	Greater than 0.05	Greater than 0.05
Result	There is a statistically significant difference between the two groups, indicating a clear effect of the laser in raising fluoride levels	No statistically significant difference	No statistically significant difference	No statistically significant difference

The results of the Mann-Whitney U test showed a significant statistical difference in fluoride ratios between the two groups (P value = 0.026) smaller than 0.05, indicating the effectiveness of using CO _2_ lasers in enhancing fluoride absorption within the tooth structure. In contrast, the remaining elements (calcium, phosphorus, carbon) did not show significant differences, although there were apparent changes in the arithmetic mean, in the rest of the descriptive statistics measures, and in the ranks of the averages ( Table 6).

## Discussion

This study evaluated the efficacy of a CO _2_ laser for enhancing fluoride uptake in dental enamel. According to existing literature, the combined application of a CO _2_ laser with fluoride varnishes or gels can potentially quadruple fluoride absorption, which may significantly improve caries prevention and reduce the required fluoride dosage or frequency of clinical applications. ^13^ While our current laboratory findings support this positive synergistic trend by demonstrating a statistically significant increase in surface fluoride concentrations (P = 0.026) via EDX analysis, these results must be interpreted within specific boundaries.

Due to the small sample size utilized in this investigation (n = 6 permanent teeth sectioned to create 12 experimental halves), this work should be appropriately characterized as a pilot study. In advanced microstructural and surface examinations using SEM and EDX, small sample sizes are frequently employed because the focus is on highly precise, localized topographic and elemental changes rather than biological variance. Furthermore, our study utilized a split-tooth design—where each tooth served as its own internal control (mesial half treated with laser + fluoride, distal half with fluoride alone)—which effectively eliminated inter-individual biological variability and enhanced the statistical power of the comparison. Nevertheless, we acknowledge the small sample size as a limitation of this laboratory model, and future large-scale quantitative trials are required to fully validate the clinical extent of this laser-induced enhancement.

In order to obtain a protective effect, a laser was used to raise the temperature of the tooth enamel to the melting point of its inorganic components or to the decomposition point of its organic components. On the other hand, non-thermal laser radiation was used after tooth fluoridation to achieve a slow fluoride release effect. This type of reaction is called laser-activated fluoride treatment. ^11^

Carbon dioxide laser irradiation of tooth enamel, within certain parameters, results in a specific temperature increase above the irreversible pulpitis threshold of 5.5 °C, ^14^ which changes the chemical composition of the tooth enamel surface and the morphological structure ^15^ and the increase may lead to permanent damage to the tooth pulp.

Excessive temperature elevation during laser application poses a risk of injury to the dental pulp. A study addressing this risk compared the thermal effects of two laser systems: a CO _2_ laser (at 1 and 2 W) and a diode laser (at 5 and 7 W). The results indicated that the mean temperature rise remained below the 5.5 °C critical threshold for pulp damage. Based on this evidence, the authors determined that both lasers could be used safely without compromising pulp health. ^16^

The absorption depth of port and ivory is 12 μm, compared to the absorption depth of water of 15 μm. ^17^

The absorption effects of tooth enamel and dentin at these depths depend on how the laser energy affects them and the laser energy depends on laser parameters such as power, mode of operation (continuous or pulsed wave mode), flow (energy density) and dose.

The continuous wave mode resulted in carbonization, dissolution and cracking of the enamel and the development of the pulse mode allowed variations by frequency and pulse duration in milliseconds (ms) and microseconds (μs). This improved the thermal control of the laser in tissue. The dose depends on the radiation method: spot radiation or sweeping motion, the number of repeated radiations. ^9^

Several hypotheses have been advanced to explain the mechanisms by which combined laser and fluoride treatment enhances resistance to enamel demineralization. A principal theory posits that laser irradiation creates micro-spaces within the enamel structure, which subsequently serve to retain fluoride ions more effectively. ^18^ These structural alterations are also believed to contribute directly to reducing enamel solubility. ^19^ The observed morphological changes on the enamel surface following the sequential application of topical fluoride and CO _2_ laser irradiation provide a rationale for the superior protective effect compared to fluoride treatment alone. ^20^ This synergistic effect is further attributed to the laser-induced conversion of hydroxyapatite to the more acid-resistant fluorapatite phase, a process accompanied by the melting and recrystallization of hydroxyapatite crystals. ^21^

Several hypotheses have been advanced to explain the mechanisms by which combined laser and fluoride treatment enhances resistance to enamel demineralization. A principal theory posits that laser irradiation creates micro-spaces within the enamel structure, which subsequently serve to retain fluoride ions more effectively. These structural alterations are also believed to contribute directly to reducing enamel solubility. The observed morphological changes on the enamel surface following the sequential application of topical fluoride and CO _2_ laser irradiation provide a rationale for the superior protective effect compared to fluoride treatment alone. This synergistic effect is further attributed to the laser-induced conversion of hydroxyapatite to the more acid-resistant fluorapatite, a process accompanied by the melting and recrystallization of hydroxyapatite crystals. ^19^

The results of previous studies showed that treating the surface of tooth enamel using a carbon dioxide laser reduces demineralization of tooth enamel compared to the control group. Studies have shown that wavelengths from 9 to 11 micrometers of CO _2_ lasers are effectively absorbed by dental hydroxyapatite, causing loss of carbonate mineral, which in turn reduces acid reactivity. ^22, 23^

The integration of lasers in preventive dentistry reflects a modern paradigm shift within the field, moving away from a traditional focus on restorative treatment and toward a philosophy of disease prevention. This approach is consistent with the recommendations of major professional bodies and is part of a wider scientific consensus advocating for less invasive caries management strategies, ^24^ Which aims to preserve tissues to the maximum extent, and in this new approach, prevention has acquired an important role that it has not played before in the past. ^11^

All experiments combining CO _2_ and fluoride laser radiation have shown better results in preventing tooth caries when compared to a single treatment. Therefore, this ‘combination therapy’ may be clinically effective, and at the same time, it may only involve moderate daily doses of fluoride and low levels of laser radiation energy, which is consistent with our current study.

## Conclusion

Within the limitations of this in vitro study, the combined application of a 10,600 –nm CO _2_ laser and sodium fluoride varnish demonstrated a significant synergistic effect, clinically translating into enhanced enamel surface resistance to acid challenges and a marked increase in fluoride retention compared to fluoride treatment alone. From a clinical perspective, these findings imply that utilizing low-energy CO _2_ laser irradiation could potentially optimize preventive protocols in pediatric dentistry and orthodontics by reducing the required frequency of fluoride applications and offering long-term enamel protection. However, because this laboratory model cannot fully replicate the complex dynamic biological environment of the oral cavity—including salivary flow, pellicle formation, and microbial biofilms—further randomized controlled clinical trials are essential to completely validate the clinical efficacy and sustainability of this laser-assisted approach.

### Recommendations

Although these results are very promising, more studies should be developed in order to test these radiation conditions under different challenges, and simulate other clinical variables and therefore, future clinical studies are recommended to confirm these results.

## Data Availability

The dataset includes raw EDX values for fluoride, calcium, phosphorus, and carbon, as well as the data used for statistical analyses and tables are It is available to the public at this link. Figshare: Comparison between the protective effect of applying sodium fluoride varnish alone and applying CO _2_ laser with it in permanent teeth. https://doi.org/10.6084/m9.figshare.31333387.v2 ^25^ The dataset includes:
• Research Data • EDx images Data are available under the terms of the CC BY 4.0 Extended data associated with this study, including EDX spectra, and detailed statistical output tables, are available in the same repository as the underlying data under a CC BY 4.0 license.
