# Supplementary material for: Effect of Combined CO₂ Laser Irradiation and Sodium Fluoride Varnish on Enamel Surface Morphology and Surface Elemental Composition: An In Vitro Split-Tooth Study
Source: F1000Res. 2026 Jul 23;15:588. Originally published 2026 Apr 19. [Version 3] doi: 10.12688/f1000research.171971.3 (PMC13560542; doi:10.12688/f1000research.171971.3)
Supplement: Peer review history

# Reviewer report: Reviewer response for version 3

**DOI:** 10.5256/f1000research.206702.r504291

**Published:** 2026-08-10

**Authors:** Sanpreet Singh Sachdev

**Affiliations:** 1 Oral Pathology and MIicrobiology, Bharati Vidyapeeth (Deemed to be University) Dental College and Hospital Navi Mumbai, Navi Mumbai, Maharashtra, India

The subsections of figures are not labelled and described. The redundant full forms of SEM and EDX are mentioned repeatedly still. Ethical approval section reveals all details about the authors' university, which is against the jurnals policy of anonymity. Tables are still not merged and presented separately. They should be merged to improve readability of the mansucript helping readers to understand the results at a glance. There is a lot of redundancy btween the methods, results and discussion sections

Is the work clearly and accurately presented and does it cite the current literature?

No

If applicable, is the statistical analysis and its interpretation appropriate?

I cannot comment. A qualified statistician is required.

Are all the source data underlying the results available to ensure full reproducibility?

No source data required

Is the study design appropriate and is the work technically sound?

No

Are the conclusions drawn adequately supported by the results?

No

Are sufficient details of methods and analysis provided to allow replication by others?

No

Reviewer Expertise:

Oral Pathology, Dental Materials, Dental Innovations, Dental Anatomy, Dentistry

I confirm that I have read this submission and believe that I have an appropriate level of expertise to state that I do not consider it to be of an acceptable scientific standard, for reasons outlined above.

# Reviewer report: Reviewer response for version 3

**DOI:** 10.5256/f1000research.206702.r500014

**Published:** 2026-08-07

**Authors:** Mays Sameer Tareq

**Affiliations:** 1 University of Technology, Baghdad, Iraq

***The Reviewer's Comments***

**Recommendation: Accept with major revision.**

***Summary:***

 The manuscript contains various grammatical mistakes and typos. A comprehensive linguistic editing is necessary.

***Weaknesses and Suggestions:***

***Abstract***

 Some experimental data need to be included in this section to give a clear insight on the results and their discussions instead of some general statements and unnecessary comparisons.

***Introduction***

1. In the introduction, if abbreviations are used, they should be consistently applied throughout the text. Also, include similar research related to your work and briefly compare their results. When stating the objective of the study, clearly mention the variable parameters. At the end of the introduction, introduce the upcoming sections: In the next section, we consider materials and methods applied to the experiment. Section 3 shows the …. In Section 4, we present the results and discussion. Section 5 illustrates the …. In the final section, acknowledgements and author contribution are mentioned.
2. The research objective should be written in a more concise and objective manner.

***Experimental Methodology,*** ***results, and discussion:***

1. What is the novelty of this work?
2. The article describes a split-tooth approach, using the mesial and distal half of the same tooth as paired experimental and control groups. This is a strong design that takes into account interindividual variability. The statistical analysis was performed with Mann-Whitney U test, which is for independent samples. Why not use a paired test (e.g. Wilcoxon signed rank test)? The principal finding of the study (p=0.026 for fluoride) may not be legitimate because of the use of an incorrect statistical test. “It is absolutely essential to re-analyze with the proper test.
3.  The study used just six teeth (n=6 per group).  The authors say in the response to reviewers that this is a “pilot study”. However, the abstract and conclusions describe the finding as if it is final. Was a formal power analysis done a prior to establish that a sample size of six was adequate to detect a clinically relevant difference? But if so, why can this study be recognized as anything more than a preliminary pilot research and why was this not clearly indicated in the title and abstract?
4.  The Abstract mentions teeth halves were "randomly assigned" to groups, however the Methods section indicates that the mesial half was always allocated to the laser+fluoride group and the distal half to the fluoride-only group. What is correct? If allocation was not randomised there is considerable possibility of systematic bias. For example, is there a structural difference between the mesial and distal surfaces that confounds the results? Please explain exactly how the allocation was made and argue why the use (or absence) of randomisation does not undermine the validity of the study.
5. The title and findings of the study boldly assert a “protective effect” and the possibility of “long-term enamel protection”. However, only the surface elemental composition was evaluated using SEM/EDX in the research, showing a rise of fluoride, calcium and phosphorus on the surface. This does not directly evaluate fluoride absorption into the enamel, or fluorapatite production or resistance to acid demineralisation. Your findings do not include a test of acid resistance (e.g., a pH cycling model). How can you make such broad claims regarding clinical effectiveness and caries prevention from this in vitro trial alone?
6. How was the varnish removed from the surface of the tooth before the SEM/EDX analysis? If it wasn't removed, are you measuring fluoride in the varnish residue and not the enamel?

 

1. The conclusion suggests that the combination treatment is “clinically translating to enhanced enamel surface resistance to acid challenges”. No acid challenge or demineralisation test was performed in this research. This is a little exaggeration of your findings. How can you square this finding with the fact that your study didn’t truly test acid resistance?
2. There is an unnecessary paragraph on the mechanisms of action in the manuscript (pages 9 and 10). More crucially, the statement "The continuous wave mode resulted in carbonisation, dissolution and cracking of the enamel... " is referenced without citation and is opposed to your own pulsed-mode laser technique. If you did not employ continuous wave mode in your study, why is this subject relevant? Please elaborate and cite.
3. Discuss the limitations of their study that they had observed during conducting this study.
4.  For future work, it is suggested that the authors explore whether the proposed system can be applied to other types of materials, or how it can be combined with other advanced technologies to improve overall performance.

***Conclusions***

 The conclusion section should be rewritten, briefly summarize and mention the main findings of all analyses, highlight the novelty of the work, and suggest potential ideas for future studies.

Is the work clearly and accurately presented and does it cite the current literature?

Yes

If applicable, is the statistical analysis and its interpretation appropriate?

Partly

Are all the source data underlying the results available to ensure full reproducibility?

Yes

Is the study design appropriate and is the work technically sound?

Yes

Are the conclusions drawn adequately supported by the results?

Partly

Are sufficient details of methods and analysis provided to allow replication by others?

Partly

Reviewer Expertise:

laser-tissue interaction. laser ablation, laser medical applications, nanotechnology. and lasers in dentistry

I confirm that I have read this submission and believe that I have an appropriate level of expertise to confirm that it is of an acceptable scientific standard, however I have significant reservations, as outlined above.

# Reviewer report: Reviewer response for version 3

**DOI:** 10.5256/f1000research.206702.r504290

**Published:** 2026-08-03

**Authors:** Vikas Singh

**Affiliations:** 1 Teerthanker Mahaveer University, Moradabad, Uttar Pradesh, India

The authors have made substantial efforts to address the methodological and interpretational concerns raised during the previous round of peer review. The revised manuscript demonstrates clear improvements in statistical methodology, methodological transparency, and interpretation of the findings. Most of the major concerns identified in my previous review have been satisfactorily addressed.

 In particular, replacing the Mann-Whitney U test with the Wilcoxon Signed-Rank test appropriately reflects the paired split-tooth design, where each tooth serves as its own control. The inconsistency regarding specimen allocation has also been resolved, and the manuscript now clearly describes the mesial halves as the experimental specimens and the distal halves as the controls. The addition of methodological details regarding fluoride varnish removal, examiner blinding, and EDX acquisition parameters has improved the reproducibility of the experimental procedures. Furthermore, the Discussion and Conclusion have been revised to appropriately acknowledge that SEM and EDX provide only topographic and elemental information and do not directly demonstrate fluorapatite formation, enhanced acid resistance, or clinical caries prevention. The inclusion of a dedicated limitations section is also appreciated.

 Although the manuscript has improved substantially, several issues remain that would further strengthen the scientific presentation.

 First, the statistical reporting remains incomplete. The Methods indicate that effect sizes (r) and 95% confidence intervals were calculated, yet these values are not fully presented in Table 6, which currently reports only Z-statistics and P-values. Reporting the actual effect sizes together with their corresponding confidence intervals would allow readers to better appreciate the magnitude and precision of the observed differences. Wherever possible, exact P-values should also be reported instead of threshold values such as "<0.001."

 Second, despite the improved discussion of limitations, the study remains based on a very small sample of six teeth (twelve paired specimens). While the split-tooth design appropriately controls for inter-individual variability and increases the efficiency of paired comparisons, the findings should continue to be interpreted as exploratory laboratory observations. The Discussion should consistently avoid language implying broader clinical effectiveness beyond what can reasonably be concluded from this pilot in vitro investigation.

 Third, although the authors have appropriately clarified that EDX measures elemental composition rather than fluorapatite formation, several statements continue to imply that increased elemental fluoride necessarily reflects enhanced fluoride uptake within enamel. Since EDX is primarily a surface elemental analysis technique and cannot distinguish between loosely retained surface fluoride, chemically incorporated fluoride, or fluorapatite formation, the manuscript would benefit from consistently referring to increased ***surface fluoride concentration*** rather than enhanced fluoride uptake unless supported by additional analytical methods.

 Finally, while statistically significant differences in elemental composition are reported, the biological significance of these differences remains uncertain. The Discussion would be strengthened by placing the magnitude of the observed elemental changes into context through comparison with previous quantitative studies and by discussing whether the observed differences are likely to be clinically meaningful.

 Overall, the revised manuscript is substantially stronger than the previous version. The principal methodological concerns raised during the earlier review-including the statistical analysis, clarification of specimen allocation, methodological transparency, examiner blinding, specimen preparation, and more cautious interpretation of the findings-have been satisfactorily addressed. The remaining issues relate primarily to reporting completeness and scientific presentation rather than fundamental methodological flaws.

 In its current form, I consider the manuscript scientifically acceptable as a pilot in vitro investigation, provided that the remaining reporting and presentation issues outlined above are addressed.

 I therefore support indexing with following **minor revisions**.

Is the work clearly and accurately presented and does it cite the current literature?

Partly

If applicable, is the statistical analysis and its interpretation appropriate?

No

Are all the source data underlying the results available to ensure full reproducibility?

Partly

Is the study design appropriate and is the work technically sound?

No

Are the conclusions drawn adequately supported by the results?

No

Are sufficient details of methods and analysis provided to allow replication by others?

Partly

Reviewer Expertise:

Public Health Dentistry, Preventive Dentistry, Oral Epidemiology, Clinical Research Methodology, Biostatistics, Evidence-Based Dentistry, Dental Materials

I confirm that I have read this submission and believe that I have an appropriate level of expertise to confirm that it is of an acceptable scientific standard, however I have significant reservations, as outlined above.

# Reviewer report: Reviewer response for version 2

**DOI:** 10.5256/f1000research.203413.r500020

**Published:** 2026-07-16

**Authors:** Vikas Singh

**Affiliations:** 1 Teerthanker Mahaveer University, Moradabad, Uttar Pradesh, India

This manuscript investigates the effect of CO₂ laser irradiation combined with topical fluoride on enamel morphology using an in vitro split-tooth design. The topic is relevant and the study has potential as an exploratory laboratory investigation. However, **several major methodological and interpretational concerns substantially reduce confidence in the validity of the findings.**

 The most important concern relates to the statistical analysis. ***The study is described as a split-tooth design in which paired observations are obtained from the same tooth, yet the analysis was performed using the Mann-Whitney U test, which assumes independent samples. *** This approach requires clear justification or reanalysis using an appropriate paired statistical method.

 In addition, the manuscript contains contradictory descriptions of specimen allocation. **The Abstract states that tooth halves were randomly assigned to the experimental and control groups, whereas the Methods indicate that the mesial half was consistently assigned to the experimental group and the distal half to the control group. **This inconsistency must be resolved because it directly affects the validity of the experimental design.

 The conclusions also extend beyond the evidence generated by the study. SEM and EDX analyses demonstrate changes in enamel surface morphology and elemental composition, but they do not directly demonstrate fluoride uptake, fluorapatite formation, increased acid resistance, or clinical caries prevention. The conclusions should therefore be restricted to the measured outcomes.

 Additional methodological details required for reproducibility should be provided, including the EDX acquisition protocol, specimen preparation, varnish removal before analysis, examiner blinding, and measurement standardization. The Discussion should better acknowledge the limitations of this pilot in vitro study and avoid claims that are not directly supported by the experimental findings.

 Minor revisions include improving the title to better reflect the measured outcomes, reducing repetition in the Results section, reporting effect sizes and confidence intervals where appropriate, consolidating descriptive tables where feasible, and correcting the formatting of several references and remaining language issues.

 Overall, although the research question is scientifically relevant, the combination of methodological inconsistencies, apparently inappropriate statistical analysis, contradictory specimen allocation, and conclusions that exceed the available evidence means that the manuscript does not currently meet the standard required for approval. Major methodological issues would need to be addressed before the work could be considered scientifically sound.

Is the work clearly and accurately presented and does it cite the current literature?

Partly

If applicable, is the statistical analysis and its interpretation appropriate?

No

Are all the source data underlying the results available to ensure full reproducibility?

Partly

Is the study design appropriate and is the work technically sound?

No

Are the conclusions drawn adequately supported by the results?

No

Are sufficient details of methods and analysis provided to allow replication by others?

Partly

Reviewer Expertise:

Public Health Dentistry, Preventive Dentistry, Oral Epidemiology, Clinical Research Methodology, Biostatistics, Evidence-Based Dentistry, Dental Materials

I confirm that I have read this submission and believe that I have an appropriate level of expertise to state that I do not consider it to be of an acceptable scientific standard, for reasons outlined above.

# Reviewer report: Reviewer response for version 2

**DOI:** 10.5256/f1000research.203413.r498588

**Published:** 2026-07-10

**Authors:** Sanpreet Singh Sachdev

**Affiliations:** 1 Oral Pathology and MIicrobiology, Bharati Vidyapeeth (Deemed to be University) Dental College and Hospital Navi Mumbai, Navi Mumbai, Maharashtra, India

The study title is scientifically inappropriate. Also, the word pilot study should be included in the title given that the sample size is vastly deficient to be considered as proper original research.

- The study aim itself is incorrect. The authors are advised to use a simple language that is easy to understand by the readers.
- Brand, city, country of the materials are required only at the first instance of mention
- Why were the root halves not randomly distributed? The manuscript discusses it as a split-tooth design where each tooth serves as its own control, but each group had the fixed root that could have confounded the results owing to differences in the anatomy.
- Expansions of the abbreviations are only needed at the first instance.
- Label both parts of the figures as a) and b) and described what is to be seen in them
- Tables are better combined in a single table for ease of readers.
- last paragraph of discussion is unclear. whether the authors are talking about a previous study or describing their overall findings? the phrase consistent with our current study, implies that earlier sentences are referring to earlier studies. References are required for those.
- The claim made in the discussion that 'The continuous wave mode resulted in carbonization, dissolution and cracking of the enamel and the development of the pulse mode allowed variations by frequency and pulse duration in milliseconds (ms) and microseconds (μs). ' Is unsubstantiated and scientifically inaccurate. Authors must review carefully all claims made when discussing the findings. 

Is the work clearly and accurately presented and does it cite the current literature?

No

If applicable, is the statistical analysis and its interpretation appropriate?

I cannot comment. A qualified statistician is required.

Are all the source data underlying the results available to ensure full reproducibility?

No source data required

Is the study design appropriate and is the work technically sound?

No

Are the conclusions drawn adequately supported by the results?

No

Are sufficient details of methods and analysis provided to allow replication by others?

No

Reviewer Expertise:

Oral Pathology, Dental Materials, Dental Innovations, Dental Anatomy, Dentistry

I confirm that I have read this submission and believe that I have an appropriate level of expertise to state that I do not consider it to be of an acceptable scientific standard, for reasons outlined above.

# Reviewer report: Reviewer response for version 1

**DOI:** 10.5256/f1000research.189644.r485241

**Published:** 2026-06-04

**Authors:** Sanpreet Singh Sachdev

**Affiliations:** 1 Oral Pathology and MIicrobiology, Bharati Vidyapeeth (Deemed to be University) Dental College and Hospital Navi Mumbai, Navi Mumbai, Maharashtra, India

The introduction states the importance of fluoride, then abruptly moves to the harmful effects of fluoride, followed by the enhancement using a laser. Nowhere is the need for the application of lasers specified. Is the normal fluoride application insufficient such that additional laser application is required?

 The introduction is majorly unformatted and needs proper structuring in paragraphs. Needs better flow of the content.

 Table 1 not required. Non-contributory to manuscript. Authors can just cite the justification for their range. SEM and EDX full forms are repeated. 

 The AI generated placeholder [“Not applicable as the study used discarded biological material”] has got retained in the manuscript after the ethical approval number. The authors probably forgot to remove it. So currently there is a reference ethical approval number and 'not applicable' statement at the same time, which are contradicting each other.

 Indicate by arrows what is to be seen in the SEM images, and also mention that in the legends. Include SEM results in the abstract too.

 Discussion makes a strong claim 'The combined application of a CO _2_ laser with fluoride varnishes or gels, which can quadruple fluoride absorption, may significantly improve caries prevention. ' The authors need to specify that such a claim is based on their results or from existing literature. If existing literature, cite the references accordingly. But in this case, the present study is not required, if it is already established that lasers enhance the binding four-fold. If based on the present study's findings, it is too strong a claim based on an in vitro study on 12 samples.

 Justification for the sample size is required, otherwise the study is just a pilot study with a small sample.

 Citations for all the mechanisms stated for action of laser are required. Currently, only one reference is cited at the end of the paragraph discussing the mechanisms.

 The discussion flow is abrupt and inconsistent, requiring overall re-writing.

 Conclusion must have a modest tone, and organized as a paragraph rather than bulleted list.

Is the work clearly and accurately presented and does it cite the current literature?

No

If applicable, is the statistical analysis and its interpretation appropriate?

I cannot comment. A qualified statistician is required.

Are all the source data underlying the results available to ensure full reproducibility?

No source data required

Is the study design appropriate and is the work technically sound?

No

Are the conclusions drawn adequately supported by the results?

No

Are sufficient details of methods and analysis provided to allow replication by others?

No

Reviewer Expertise:

Oral Pathology, Dental Materials, Dental Innovations, Dental Anatomy, Dentistry

I confirm that I have read this submission and believe that I have an appropriate level of expertise to state that I do not consider it to be of an acceptable scientific standard, for reasons outlined above.

# Response

**Published:** 2026-06-06

**Authors:** Abdulrhman Alkhaled

**Affiliations:** Damascus University Faculty of Dentistry, Damascus, Damascus Governorate, Syria

**تاResponse:**

 We sincerely thank the reviewer for this constructive critique.

 We agree that the rationale for introducing laser therapy was not sufficiently justified. In the revised manuscript (Introduction, Paragraph X), we have restructured the text to bridge this gap. We have clearly specified that while conventional fluoride application is highly effective, its clinical longevity is limited due to dissolution in oral fluids and sub-optimal deep-tissue uptake. CO2 laser irradiation is introduced not because normal fluoride is "insufficient," but as a synergistic modality to alter the enamel surface topography and thermal properties, thereby enhancing the retention, conversion, and depth of fluoride penetration into fluorapatite.

 The Introduction has been thoroughly rewritten and restructured into logical, well-demarcated paragraphs to ensure a smoother transition from the background of dental caries prevention to the specific objective of this study.

 Table 1 has been removed from the manuscript, and the laser parameters have been integrated textually within the Methodology section along with the appropriate justification and references. Additionally, all redundant full forms of SEM and EDX have been corrected, keeping the full term only upon first mention.

 The contradictory placeholder was inadvertently left during the final drafting process. It has been completely removed in Version 2. The official ethical approval number [Insert Number] stands alone as the correct authorization for this study.

 In the revised manuscript, we have added distinct arrows to the SEM micrographs to explicitly point out structural changes (e.g., enamel melting, micro-cracks, and crystal precipitation). The figure legends have been updated to explain these indicators. Furthermore, a concise summary of the SEM and EDX findings has been incorporated into the Abstract.

 This specific statement ("quadruple fluoride absorption") was cited from existing literature to highlight the maximum potential described in historical protocols, rather than being a direct finding of our study. We agree that extrapolating this as a definitive claim based on our in vitro sample size (n=12 treatment sites) is scientifically overreached. In Version 2, we have rephrased this section to adopt a more modest and objective tone, clearly differentiating between historical literature findings and our own current laboratory observations. Proper citations have been added

 In in vitro studies employing advanced surface analyses like SEM and EDX, small sample sizes are frequently utilized due to the high precision, micro-destructive nature, and qualitative depth of the evaluation, which focus on structural topography rather than biological variance. However, we acknowledge the reviewer's perspective that without a formal prior power analysis, this work should be characterized appropriately. We have now included a paragraph in the Discussion acknowledging this as a pilot investigation and have explicitly listed the sample size as a limitation, which paves the way for future large-scale quantitative evaluations.

**Response: **We thank the reviewer for pointing this out. We have thoroughly revised the Discussion section to ensure a logical and scholarly flow. Multiple missing citations validating the photothermal and structural mechanisms of the 10,600\text{ nm} CO_2 laser on dental enamel have been integrated into the relevant paragraph. The entire section has been streamlined to prevent abrupt transitions.

 The bulleted conclusion has been transformed into a cohesive, well-structured paragraph. The tone has also been modulated to be highly conservative and strictly reflective of our in vitro findings, using hedging verbs such as "suggests" and "within the limitations of this laboratory study."

# Reviewer report: Reviewer response for version 1

**DOI:** 10.5256/f1000research.189644.r480471

**Published:** 2026-05-19

**Authors:** Alhan Ahmed Qasim

**Affiliations:** 1 University of Baghdad, Baghdad, Baghdad Governorate, Iraq

Greetings :

 1. Introduction: It needs to highlight the important of enamel protection role against acid dissolution.

 paragraph 3 " Recent epidemiological trends indicate .............." the reference is old ; attention should be paid to this.

 2. Discussion: the results of this study need further clarification and comparison  with other studies, and it is preferable recent.

 The authors did not mention the limitations of this study.

 3. Conclusion: second point"  Laser therapy can be an alternative or synergistic to topical fluoridation to prevent tooth enamel caries with a longer-lasting effect" according to the result of this study laser is synergistic to topical fluoride application to increase fluoride uptake by enamel surface.

Is the work clearly and accurately presented and does it cite the current literature?

Partly

If applicable, is the statistical analysis and its interpretation appropriate?

Yes

Are all the source data underlying the results available to ensure full reproducibility?

Yes

Is the study design appropriate and is the work technically sound?

Yes

Are the conclusions drawn adequately supported by the results?

Partly

Are sufficient details of methods and analysis provided to allow replication by others?

Yes

Reviewer Expertise:

Preventive Dentistry

I confirm that I have read this submission and believe that I have an appropriate level of expertise to confirm that it is of an acceptable scientific standard.

## References

[1] : CO2 Laser and Topical Fluoride Therapy in the Control of Caries Lesions on Demineralized Primary Enamel. *The Scientific World Journal* .2015;2015(1) : 10.1155/2015/547569 10.1155/2015/547569 PMC438566925874248
